# Time-course analysis of cardiac and serum galectin-3 in viral myocarditis after an encephalomyocarditis virus inoculation

**DOI:** 10.1371/journal.pone.0210971

**Published:** 2019-01-23

**Authors:** Kei Noguchi, Hiroyuki Tomita, Tomohiro Kanayama, Ayumi Niwa, Yuichiro Hatano, Masato Hoshi, Shigeyuki Sugie, Hideshi Okada, Masayuki Niwa, Akira Hara

**Affiliations:** 1 Department of Tumor Pathology, Gifu University Graduate School of Medicine, Gifu, Japan; 2 Department of Biochemical and Analytical Sciences, Fujita Health University Graduate School of Health Sciences, Aichi, Japan; 3 Department of Pathology, Asahi University Murakami Memorial Hospital, Gifu, Japan; 4 Department of Emergency and Disaster Medicine, Gifu University Graduate School of Medicine, Gifu, Japan; 5 Medical Science Division, United Graduate School of Drug Discovery and Medical Information Sciences, Gifu University, Gifu, Japan; University of Miami School of Medicine, UNITED STATES

## Abstract

Galectin-3 is a β-galactoside-binding lectin which is important in cell proliferation and apoptotic regulation. Recently, serum galectin-3 has been shown to have prognostic value as a biomarker in heart failure. Encephalomyocarditis virus (EMCV) can cause severe myocarditis, congestive heart failure and dilated cardiomyopathy as well as encephalitis in various animals including mice. The pathophysiological role of galectin-3 in acute myocarditis following viral infection is not fully understood. The goal of this study is to determine the cardiac localization and the time-course of galectin-3 expression in heart failure after viral inoculation with EMCV. At 12, 24, 48, 96 hours, 7 and 10 days after intraperitoneal EMCV inoculation, animals were examined histologically and analyzed for the expression of galectin-3 and Iba1. Galectin-3 was up-regulated in degenerated fibrotic lesions of cardiac tissues 96 hours after viral inoculation and were followed by myocardial fibrosis. At the same time, Iba1 positive macrophages were observed within the inflammatory sites. A time-course correlation between the number of galectin-3 positive cells and the cardiac area of degenerated fibrotic lesions was detected—serum galectin-3 increased at 96 hours and correlated well with the number of cardiac galectin-3 positive cells. Our results indicate that galectin-3 expression may be a useful biomarker of cardiac fibrotic degeneration in acute myocarditis following viral infection. In addition, measuring serum galectin-3 levels might be an early diagnostic method for detecting cardiac degeneration in acute myocarditis.

## Introduction

Galectins are a family of beta-galactoside-binding lectins. One member of this family, galectin-3, binds glycoconjugates and IgE on mammalian cell surfaces.[1] Galectin-3 is expressed by various inflammatory cells including neutrophils, eosinophils, mast cells, macrophages, and histiocytes, which are tissue-based cells of the mononuclear phagocytic system.[2] Although galectin-3 is predominantly present as a cytosolic protein, it is also expressed on cell surfaces and secreted into plasma by various cells.[3] It has been shown that galectin-3 plays an important role in diverse physiological functions such as cell growth, apoptosis and mRNA splicing, as well as acting as a local inflammatory mediator in pathological conditions.[4]

Recent studies suggest that myocardial galectin-3 could be a marker for both cardiac inflammation and fibrosis, depending on the specific pathogenesis of human heart failure.[5–7] Therefore, galectin-3 may be a novel candidate biomarker for diagnosis, analysis and prognosis of various cardiac diseases, including heart failure.[8] Furthermore, some peptides, such as N-acetyle-aspartyl-lysyl-proline and N-acetyl-d-lactosamine, have been reported to prevent galectin-3-related cardiac inflammation, fibrosis, remodeling and dysfunction.[9, 10] This suggests that galectin-3 levels may correlate with the extent of disease progression, and, moreover, galectin-3 may be a therapeutic target for the treatment of heart failure. However, there are no reports investigating time-course of the histological localization and expression of galectin-3 and the correlation with its blood concentration in myocarditis.

Galectin-3 and macrophages have been suggested as major players driving acute inflammation and chronic fibrosis in many diseases. Ionized calcium binding adaptor molecule 1 (Iba1) is a 17-kDa protein that is specifically expressed in macrophages/microglia and is upregulated during the activation of these cells. Iba1, as a microglia/macrophage-specific calcium-binding protein, can be used to assess the relationship between galectin-3 and macrophages by using immunofluorescent staining. [11] [12]

Encephalomyocarditis virus (EMCV) is a small single-stranded RNA virus that can cause acute myocarditis in various animals including mice.[13] EMCV infection in mice is an established model for viral myocarditis, dilated cardiomyopathy and congestive heart failure.[14] In addition to myocarditis, EMCV can cause acute encephalitis in many mammalian species.[13] Our previous study[15] demonstrated that galectin-3 is up-regulated in early stages of degenerative lesions of brain areas, including cerebellum, hippocampus, thalamus and cerebral hemisphere. Interestingly, a few galectin-3 positive cells were detected in cerebellum microlesions during the immediate-early phase of encephalitis—as early as 48h –after EMCV inoculation. This findings indicate that galectin-3 expression may be a pivotal mediator between viral infection and encephalitis-induced neuronal degeneration in central nervous system, and that detection of galectin-3 might be an early diagnostic method for localized neuronal degeneration after virus infection.

We now hypothesize that analogous to viral-induced neuronal degeneration, increased galectin-3 levels may also be a diagnostic biomarker for early myocardiac lesions. To test this hypothesis, we investigated the time-course of changes of cardiac and serum galectin-3 in the EMCV-infected mouse myocarditis model. We observed that galectin-3 expression may be a pivotal mediator of cardiac fibrotic degeneration in early phase of myocarditis following EMCV infection, and that detection of serum galectin-3 might be an early diagnostic biomarker for cardiac degeneration in acute myocarditis after virus infection.

## Methods

### Mice

C57BL/6J wild type male mice were purchased from Japan SLC Inc., Hamamatsu Japan. The δ-SG KO (δ-sarcoglycan null B6.129-*Sgcd*^*tm1Mcn*^/J) mice were purchased from the Jackson Laboratory (Bar Harbor, ME) for the dilated cardiomyopathy (DCM) model of mice. The mice were housed in an isolated room on a 12 h light/dark cycle (8:00 am/8:00 pm) at 22 °C, and were given free access to food and water.

### Ethics statement

This study was carried out in strict accordance with the recommendations in the Guide for the Care and Use of Laboratory Animals of the Gifu University. The protocol was approved by the Committee on the Ethics of Animal Experiments of Gifu University (30–21). All surgery was performed under isoflurane anesthesia, and all efforts were made to minimize suffering.

### Viral inoculation

A myocardial variant of EMCV was generously provided by Dr. Seto (Keio University, Tokyo, Japan). The virus was stored at -80°C in Hanks’ balanced salt solution with 0.1% BSA until use. Male mice at 6 weeks of age were inoculated intraperitoneally with 500 plaque forming units (pfu) of EMCV in 0.1 mL of saline. The mice after inoculation were housed in an isolated room on a 12 h light/dark cycle (8:00 am/8:00 pm) at 22 °C, and were given free access to food and water. The day of virus inoculation was defined as day 0 in the current study.

### Tissue preparation

At 12, 24, 48, 96 hours, 7 and 10 days after viral inoculation, animals were perfused transcardially with physiological saline and then with phosphate-buffered 10% formalin. Control animals were used as 0 h samples. Hearts were removed and processed for paraffin embedding. Three micrometer minor axis sections of left ventricular were cut, mounted on slides and then used for conventional hematoxylin and eosin (H&E) staining and Azan staining for assessment of cardiac fibrosis.

### Immunohistochemistry

Anti-mouse galectin-3/Mac2 (galectin-3) [Rat IgG, 14–5301] was purchased from eBioscience Co.,Ltd. (San Diego, USA). Anti-Iba1 antibody [rabbit IgG, 019–19741] was purchased from Wako Pure Chemical (Osaka, Japan). The paraffinized sections were blocked to endogenous peroxidase activity by incubation in distilled water containing 3% hydrogen peroxide for 5 min. Antigen retrieval was performed, using a 0.01 M citrate buffer (pH 6.0) for both anti-galectin-3 and anti-Iba1 antibodies by the Pascal heat-induced target retrieval system (DAKO). Non-specific binding sites were blocked in 0.01 M phosphate-buffered saline (PBS), pH 7.4 containing 2% bovine serum albumin (BSA; Wako Pure Chemical, Osaka, Japan) for 30 min. Anti-galectin-3 and anti-Iba1 antibodies used at a dilution of 1:100, 1:500, respectively, in 2% BSA/PBS were added to the slides and incubated overnight at 4°C. Galectin-3 and Iba1 were detected with biotinylated anti-rat IgG (1:200, DAKO E0468), biotinylated anti-rabbit IgG (1:250, KPL-16-15-06) for 60 min, respectively, followed by incubation with avidin-coupled peroxidase (Vectastain ABC kit, Vector Laboratories) for 30 min. The peroxidase binding sites were detected by staining with 3,3’-diaminobenzidine (DAB) in 50 mM Tris-EDTA buffer. Finally, counterstaining was performed using Mayer’s hematoxylin.

### Immunofluorescent staining

The deparaffinized sections were heated in 0.01 M citrate buffer (pH 6.0) using the Pascal heat-induced target retrieval system for antigen retrieval. Non-specific binding sites were blocked in 0.01 M PBS, pH 7.4 containing 2% bovine serum albumin for 60 min, and then anti-galectin-3 and anti-Iba-1 antibodies used at a dilution of 1:100, 1:500 respectively, in 2% BSA/PBS were added to the slides and incubated overnight at 4°C. FITC-conjugated swine anti-rat IgG (1:300, Jackson Immunoresearch) and rhodamine (TRITC)-conjugated goat anti-rabbit IgG (1:100, DAKO) were used as secondary antibodies for 60 min at room temperature to visualize the fluorescent signal. After washing with PBS, the cell nuclei were stained with 4’6-diamino-2-phenylindole (DAPI) for 5 min at room temperature. Target cells were visualized on an Olympus BX-53 fluorescence microscope and DP80 camera (Olympus, Tokyo, Japan).

### Measurement of galectin-3 level in serum

The quantitative determination of galectin-3 was performed in the sera of mice that were inoculated with EMCV. Blood samples were collected at each time point from the inferior vena cava aseptically in coagulated tubes. The blood samples were centrifuged at 1,000 rpm at 4°C for 15 minutes, and then serum was collected and stored at 4°C until analysis. Serum levels of galectin-3 were measured using enzyme-linked immunosorbent assay (ELISA), according to the recommendations of the manufacturer (Abcam, Cambridge, UK).

### Statistical analyses

All statistical analyses were performed with EZR (Saitama Medical Center, Jichi Medical University, Saitama, Japan), which is graphical user interface for R (The R Foundation for Statistical Computing, Vienna, Austria). More precisely, it is a modified version of R commander designed to add statistical functions frequently used in biostatistics.[16] Data were expressed as the mean + S.E.M. and were analyzed by one-way analysis of variance (ANOVA) to determine significant differences between groups. The criterion for statistical significance was a *P* value of <0.05.

## Results

### Galectin-3 expression in degenerated fibrotic lesions of cardiac tissues

**Fig 1:**
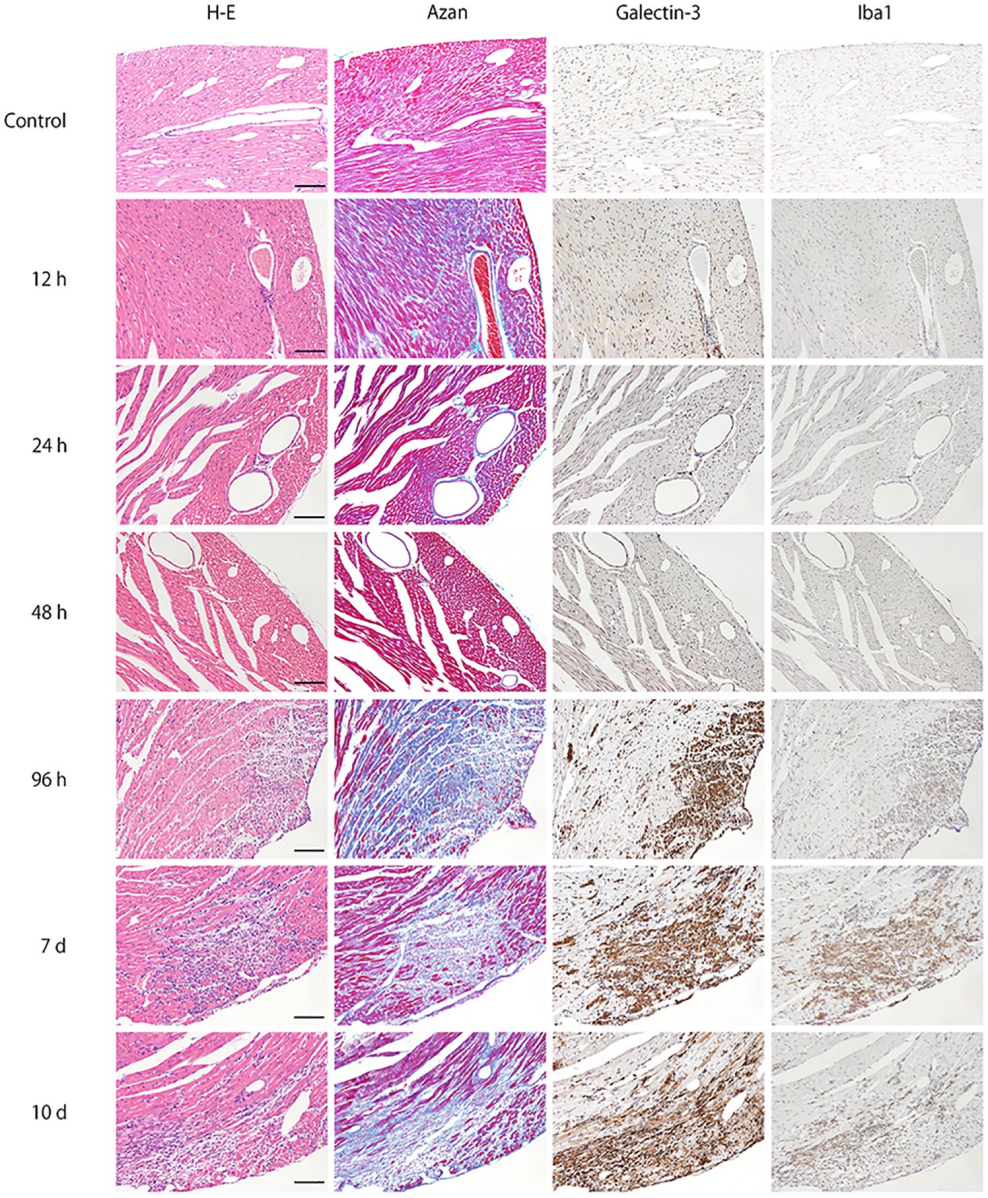
Time-course of representative microphotographs of H&E staining, Azan staining for fibrotic lesions, and immunohistochemistry for galectin-3 and Iba1 in heart tissues at 0, 12, 24, 48, 96 hours, 7 and 10 days after intraperitoneal inoculation of EMCV. For both H&E and Azan staining, notable changes was not observed until 48 hours after EMCV inoculation. Infiltration of inflammatory cells and fibrosis was observed 96 hours after inoculation. Inflammation and fibrosis peaked at 7 days after inoculation. At 0, 12 and 24 hours, there were no galectin-3 and Iba1-positive cell in heart tissues. At 48 hours after EMCV inoculation, interstitial infiltration of a few cells was detected by both galectin-3 and Iba1 immunohistochemistry, and this infiltration was greater at 96 hours. It peaked on day 7 and was observed until day 10. Scale bar = 100 μm.

Time-course of the morphological changes (H&E staining), galectin-3 positive cell numbers, the degree of the fibrosis, the serum level of the galectin-3, the inflammation score in the cardiac tissues after virus inoculation is summarized in Table 1. Representative photomicrographs of conventional H&E staining, Azan staining for fibrotic lesions, and immunohistochemistry for galectin-3 and Iba1 in heart tissues at 0, 12, 24, 48, 96 hours, 7 and 10 days after EMCV inoculation (n = 5 at each time point; total n = 30) are shown in Fig 1. Notable changes were not observed until 48 hours after EMCV inoculation, in both H&E staining and Azan staining. Infiltration of inflammatory cells in myocardial tissues was observed at 96 hours after inoculation. Inflammation and fibrosis peaked at 7 days after inoculation. In immunostaining, there were no galectin-3 and Iba1-positive cells in heart tissues at 0, 12 and 24 hours. At 48 hours after EMCV inoculation, interstitial infiltration of a few cells were detected by both galectin-3 and Iba1 immunohistochemistry. An increased number of positive cell infiltration was visible at 96 hours, with numbers peaking by 7 days and was observed until 10 days. The localization of galectin-3 was very close to that of Iba1, which indicates that galectin-3 positive cells are macrophages or histiocytes. This finding was confirmed by immunofluorescence staining.

**Table 1:** Time course of the morphological changes (HE staining), galectin-3 positive cell numbers, the degree of the fibrosis, the serum level of the galectin-3 in the cardiac tissues after virus inoculation.

Time course	Galectin-3 positive cells	Fibrosis	Serum level	Inflammation
Cont. (n = 5)	2±1	0	27±4	0
12h (n = 9)	3±1	0		0
24h (n = 8)	3±2	0	26±1	0
48h (n = 8)	8±4	1±2	29±7	0.36±0.50
96h (n = 5)	132±65	9±4	82±15	1.71±0.76
7d (n = 5)	349±87	30±7	123±32	2.6±0.54
10d (n = 2)	320±86	38±4		3±0

Cell numbers of immunoreactive galectin-3 in cardiac tissue were counted (as cell numbers/mm^2^). The degree of myocardial fibrosis was quantified as percentage of fibrotic area to total myocardial tissue in each time-course of Azan staining. The serum level of galectin-3 were measured by ELISA (ng/mL). Inflammation response score was evaluated grade 0 to 3.

### Immunofluorescence staining of galectin-3 and Iba1

**Fig 2:**
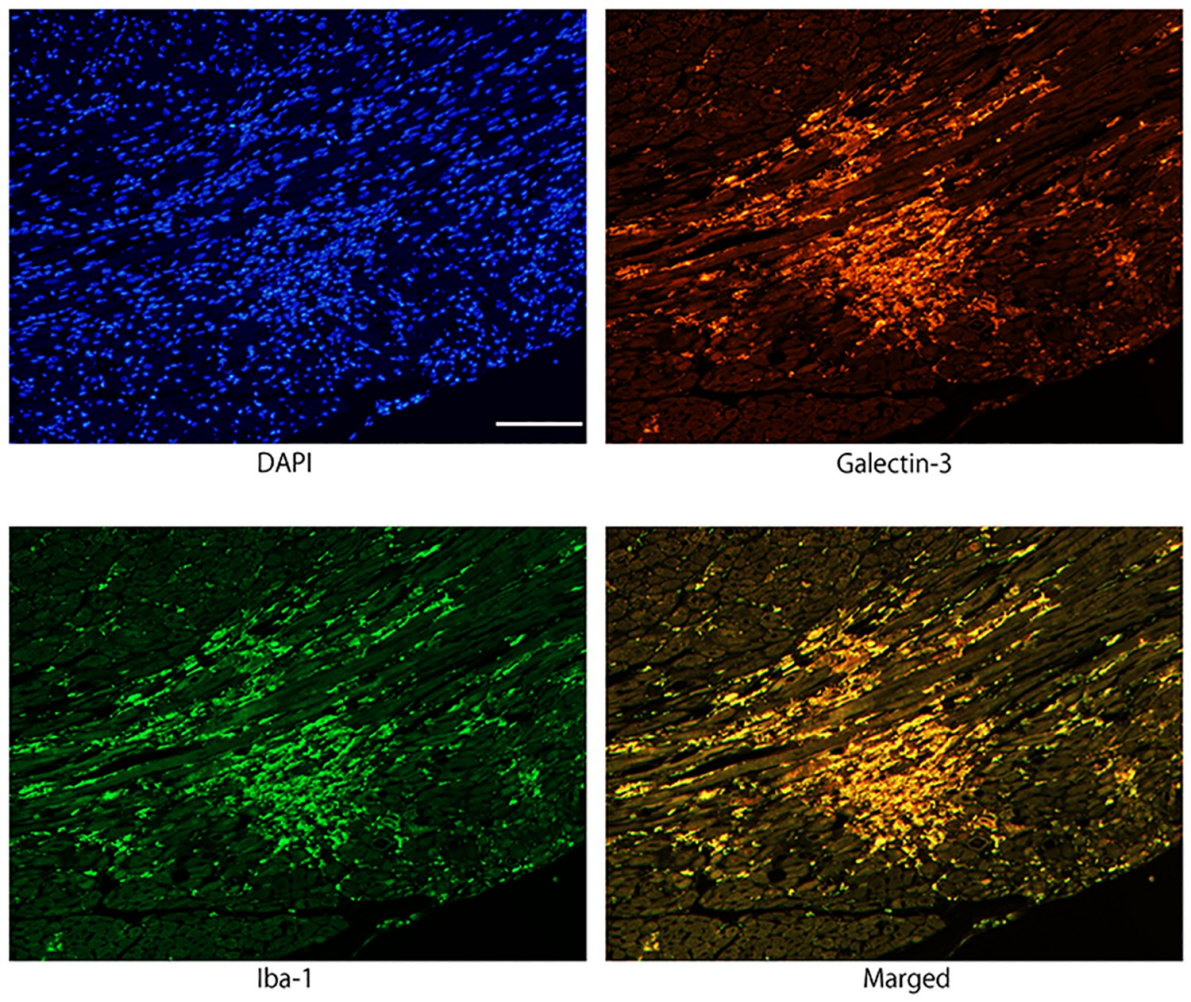
Immunofluorescence images confirming the co-localization of galectin-3 and Iba1 immunoreactivity in activated macrophages/histiocytes in myocarditis lesion 96 hours after EMCV inoculation. Galectin-3 and Iba1 were expressed in the same cells of merged photograph, indicating that infiltrating galectin-3 positive cells are macrophages. Scale bar = 100 μm.

To investigate the myocardial infiltrating cells expressing galectin-3, immunofluorescence staining for Iba1 and galectin-3 was performed in myocarditis lesion 96 hours after EMCV inoculation. Co-localization of Iba1 and galectin-3 immunoreactivity were demonstrated in the same cells, indicating that infiltrating galectin-3 positive cells are activated macrophages/histiocytes (Fig 2).

### Association of myocardial galectin-3 expression with myocardial fibrosis

**Fig 3:**
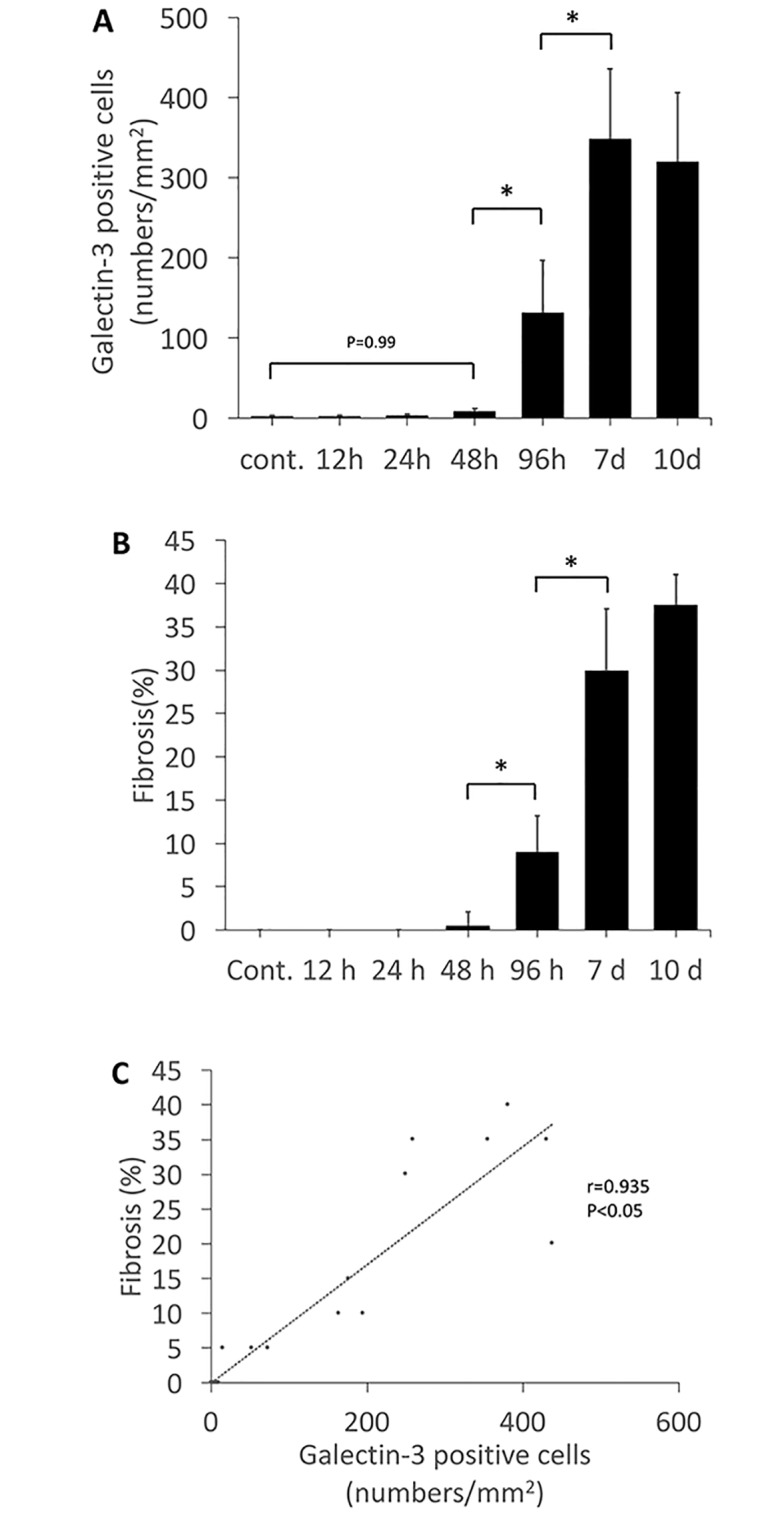
Quantification and association between the galectin-3 positive cell number and the degree of fibrosis. A, The number of galectin-3 positive cells (determined by immunohistochemistry) showing myocardial infiltration was counted at each time point. B, The degree of myocardial fibrosis (determined by of Azan staining) was quantified as percentage of fibrotic area to total myocardial tissue at each time point. C, Association between galectin-3 positive cell number and degree of fibrosis. The galectin-3 positive cell numbers were positively correlated with the degree of myocardial fibrosis. *Statistically different from each group (p < 0.05), determined by ANOVA.

The number of galectin-3 positive cells showing myocardial infiltration was counted per mm^2^ of the left ventricular tissue. [5] The galectin-3 positive cells were counted during each time-course of galectin-3 immunohistochemistry in mouse myocarditis induced by EMCV (Fig 3A). The evaluation of inflammatory response was graded as 0 = none, 1 = mild, 2 = moderate, and 3 = severe change, in accordance with the previous literature [17] (S1 Fig). The degree of myocardial fibrosis was quantified as percentage of fibrotic area to total left ventricular tissue at the largest area of the minor heart axis during each time-course of Azan staining (Fig 3B). [18] These evaluations were performed by two skilled pathologists. The galectin-3 positive cell number increased slightly at 48 hours and was followed by myocardial fibrosis. The increase of the galectin-3 positive cell numbers and the fibrotic percentage was large at 96 hours, and peaked at 7 days after virus inoculation. Galectin-3 positive cell numbers were positively correlated with the degree of myocardial fibrosis. (Fig 3C).

### Association of serum galectin-3 level with myocardial fibrosis

**Fig 4:**
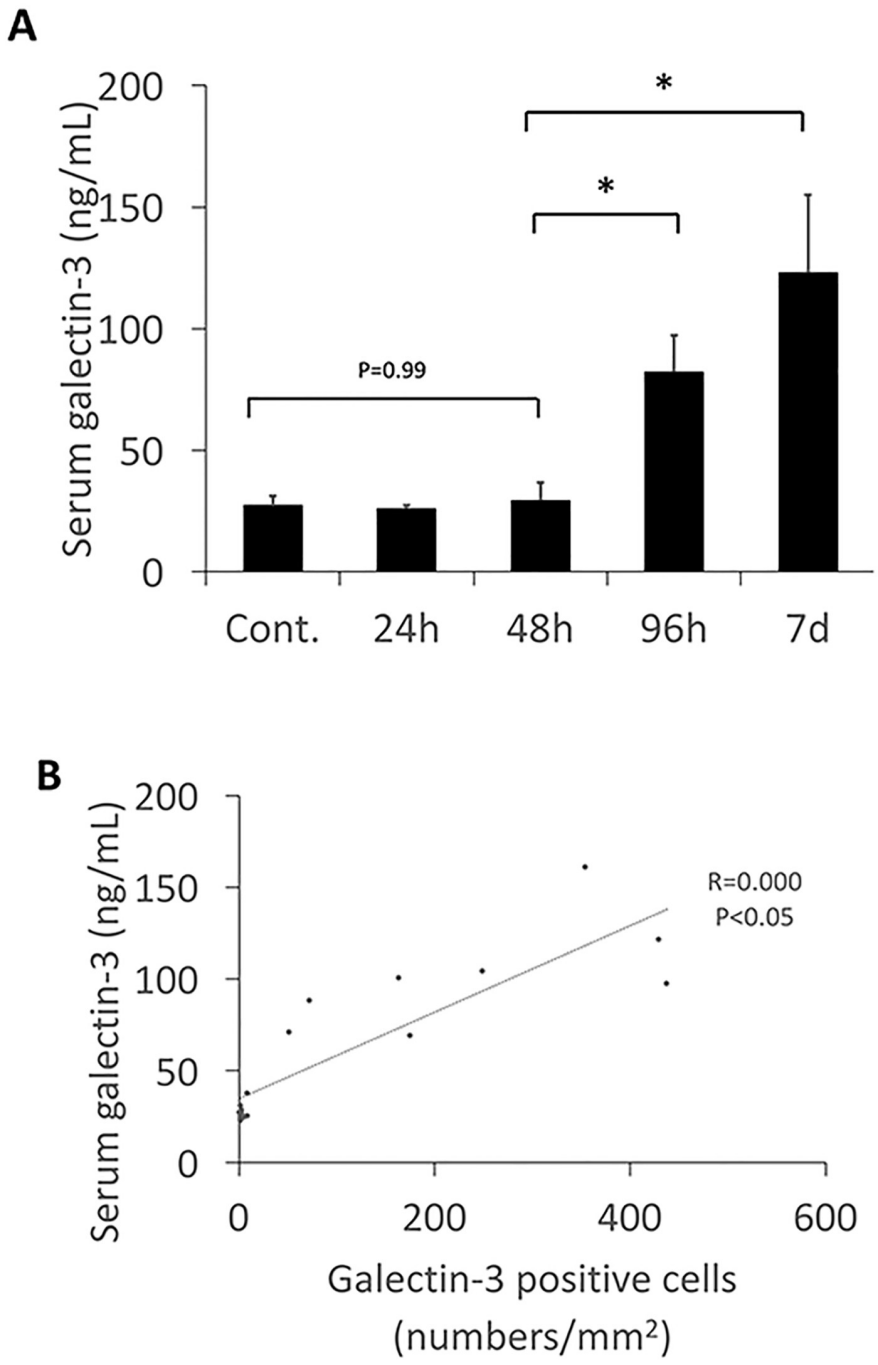
A, The serum level of galectin-3. Serum level of galectin-3 increased markedly 96 hours after virus inoculation, and continued to increase until 7 days. Data suggest there is an association between serum galectin-3 level and galectin-3 positive cell numbers or fibrosis in the cardiac tissues. *Statistically different from each group (p < 0.05), determined by ANOVA. B, Association between galectin-3 positive cell number and serum levels of galectin-3. The galectin-3 positive cell numbers were positively correlated with serum levels of galectin-3. *Statistically different from each group (p < 0.05), determined by ANOVA.

Serum levels of galectin-3 were below 30 ng/mL at 24, 48 hours after virus inoculation and was unchanged from the control. However, at the 96 hour after virus inoculation serum levels of galectin-3 had increased, and continued to increase until 7 days. The increasing of serum level of galectin-3 was similar to the number of galectin-3 positive cells and the myocardial fibrosis (Fig 4A and 4B). These data suggest that there is an association between serum galectin-3 levels and galectin-3 positive cell numbers, and the degree of myocardial fibrosis.

### Galectin-3 expression in the dilated cardiomyopathy murine model

**Fig 5:**
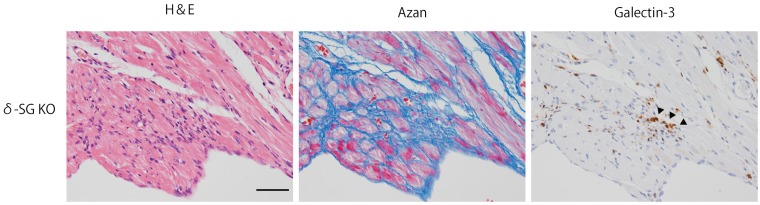
Immunohistochemistory of galectin-3 in the cardiac tissue of δ-SG KO mice which is the model of dilated cardiomyopathy (DCM). The infiltration of galectin-3 positive cells were shown. Arrows indicate galection-3 postitive cells. Scale bar = 50 μm.

We conducted additional experiments to confirm whether the present finding of galectin-3 expression is unique to patients with viral myocarditis or common to other kind of heart failure. We analyzed the dilated cardiomyopathy (DCM) model of mice. Their cardiac tissues in twelve-weeks-old male δ-SG KO (n = 2) were assessed by immunohistochemistry of galectin-3. We observed galectin-3 positive cells infiltration in this second heart failure model (Fig 5).

## Discussion

This is the first study to analyze the relationship between cardiac localization and serum level of galectin-3 in an animal model for myocarditis after a virus inoculation. Galectin-3 positive cell infiltration was visible as early as 48 hours after EMCV inoculation, with expression of galectin-3 increasing further by 96 hours after the infection, most markedly in degenerated fibrotic lesions of cardiac tissues. Positive cells peaked 7 days and were observed until 10 days after EMCV inoculation. The galectin-3 positive cell numbers correlated positively with the degree of myocardial fibrosis (confirmed by Azan staining), and, importantly, the number of galectin-3-positive infiltrating cells and the degree of myocardial fibrosis were correlated with the level of serum galectin-3. These results indicate that galectin-3 expression may be a key mediator of cardiac fibrotic degeneration in acute myocarditis following viral infection.

Sharma et al.[19] examined the source and distribution of galectin-3 in the rat myocardium in failing versus functionally compensated hearts from hypertensive Ren-2 transgenic rats by immunohistochemistry for galectin-3 protein and in situ hybridization for galectin mRNA. Their result indicated that galectin-3, a macrophage-derived mediator, was detected in areas of fibrosis, and that galectin-3 was co-localized with macrophage-specific staining. These macrophages strongly expressed MHC-II antigen as well, indicating that these cells plays an active role as antigen presentation. Thus, these finding suggest that galectin-3 induces cardiac fibroblast proliferation, collagen deposition, and ventricular dysfunction.

Liu et al.[10] hypothesize that galectin-3 induce cardiac inflammation, remodeling and dysfunction, and that these effects are mediated by the transforming growth factor (TGF)-β/Smad 3 signaling pathway. After injecting galectin-3 into the pericardial sac of rats for 4 weeks and measuring collagen deposition in the heart by histological and immunohistochemical staining, they observed that both the number of macrophages in the myocardium and the content of left ventricular collagen increased significantly in the galectin-3-injected animal group compared with vehicle-treated group. These studies implicate galectin-3, which is secreted by activated macrophages, in mediating profibrotic processes in rodent models of myocarditis-induced heart failure.

Of note, in a recent study [20] C3H/HeJ mice infected with coxsackievirus B3 (CVB-3) and depleted of macrophages (by liposome-encapsulated clodronate treatment) exhibit reduced acute myocarditis and chronic fibrosis compared with infected untreated mice, despite the former having higher viral titres. Increased galectin 3 transcriptional and translational expression levels correlated with CVB3 infection in macrophages and in non-depleted mice. Disruption of the galectin 3 gene did not affect viral titres but reduced acute myocarditis and chronic fibrosis compared with C57BL/6J wild-type mice. Similar results were observed after pharmacological inhibition of galectin 3 with N-acetyl-D-lactosamine in C3H/HeJ mice. This study only focused on acute myocarditis and chronic fibrosis, but there have been no analyses of time-course of galectin-3 expression in myocarditis-induced heart failure in animal models. A time-course study could provide insight into the processes and mechanisms of myocarditis-induced heart failure. Therefore, we conducted such a time-course study and observed that galectin-3 was up-regulated in degenerated fibrotic lesions 96 hours after viral inoculation, an early phase of viral myocarditis. We also observed that galectin-3 expressing cells in such degenerated fibrotic lesions were activated macrophages labelled by macrophage-specific immunofluorescent staining. These results support the hypothesis that galectin-3 is secreted by activated macrophages and mediates profibrotic processes in myocarditis-induced heart failure.[5, 19, 21] Furthermore, our results revealed that infiltration of galectin-3 positive cells and myocardial fibrosis started as early as 48 hours after virus inoculation and was completed by 7 days. At the same time, the serum galectin-3 level began to rise, and corresponded to the positive cell numbers and fibrosis. Thus, our results show that detection of galectin-3 might be an early diagnostic method for myocardial degeneration after virus infection.

Cardiac fibrosis and dysregulation of myocardial extracellular matrix remodeling is a key pathological feature of any cardiovascular disease.[22, 23] The progression of cardiac fibrosis is highly linked to heart failure and is associated with poor outcome in patients with cardiovascular disease.[24] On the other hand, several studies have revealed that plasma level of galectin-3 is associated with mortality and risk of heart failure with reduced ejection fraction in the general population.[25–29] These results indicate that plasma level of galectin-3 is a potential novel biomarker for cardiac fibrosis and cardiac remodeling in case of heart failure.

Besler et al.[5] tested the hypothesis that galectin-3 can be a marker for both cardiac inflammation and fibrosis, and also investigated if circulating level of galectin-3 could reflect endomyocardial galectin-3 expression. They conducted prospective cohort study of human patients with heart failure. Patients were divided into two groups to separate patients with non-ischemic, non-inflammatory dilated cardiomyopathy (DCM) (<14 leucocytes/mm^2^) and patients with inflammatory dilated cardiomyopathy (iCMP) (>14 leucocytes/mm^2^). The levels of galectin-3 were analyzed with histological features such as cardiac fibrosis and inflammatory cell infiltration in endomyocardial biopsies of patients. It was revealed that plasma galectin-3 level did not correlate with myocardial galectin-3 expression or left ventricular fibrosis neither in patients with DCM, nor in those with iCMP, whereas a positive correlation between plasma galectin-3 level and inflammatory cell numbers on endomyocardial biopsy was observed in only patients with iCMP. They concluded the plasma level of galectin-3 did not parallel endomyocardial galectin-3 expression or cardiac fibrosis by using endomyocardial biopsies of human patients.

Endomyocardial biopsy is valuable and useful in making definite diagnoses in human diseases such as myocarditis and secondary cardiomyopathies, which are often difficult to diagnose by imaging alone.[30] Despite the continuous advancements in diagnostic and therapeutic options, histologic examination remains the gold standard for cardiac diagnosis such as myocarditis and secondary cardiomyopathies like cardiac fibrosis.[31] Although human material obtained by such procedures is useful for some kinds of research, by its nature there many variables in human biopsy material, unlike those obtained from experimental animals. Human materials are usually obtained under differing conditions, such as various periods between biopsy and processing time, as well as being taken from patients with variety of disease onset or severity. In contrast, the time-course study in our preclinical model takes advantage of standardized conditions, enabling us to observed clear correlations between degree of fibrotic lesion and serum level of galectin-3.

Our results revealed that the serum level of galectin-3 reflect endomyocardial galectin-3 expression or cardiac fibrosis by using time-course animal model for histological materials, which contrasts with previous studies indicating a negative correlation in human endomyocardial biopsies. It is possible that this difference is due to a wide variability in collection and disease stage in these earlier clinical studies. Since our data clearly indicate that serum galectin-3 might be an early diagnostic method for cardiac degeneration of acute myocarditis in our preclinical mouse model, this warrants further studies to investigate whether these findings also apply for cardiac fibrosis in humans.

It should be noted that there are several limitations in the present study. First, as with any animal model, it is an extrapolation when considering relevance to myocarditis in humans. Second, EMCV can not only cause myocarditis but also encephalitis, so in a future study we will examine if encephalitis *per se* affects serum galectin-3 levels. While distinguishing galectin-3 produced in the brain from that in the heart may be difficult, galectin-3 from the cardiac tissue is likely to predominate since the blood-brain barrier would markedly limit the migration of galectin-3 produced in the brain into the blood. Thus, the contribution of encephalitis in the early phase of EMCV infection might be minimal or not detectable. Third, it is still an open question as to whether the present findings are unique to patients with viral myocarditis as opposed to myocarditis with other etiologies. However, we also conducted experiments in the murine model of DCM and observed galectin-3 positive cells infiltration, suggesting that our findings may be generalizable to other forms of heart failure associated with galectin-3.

In conclusion, our results demonstrated that galectin-3 was up-regulated in the early phase of viral myocarditis and followed by myocardial fibrosis. Galectin-3 positive cells were recognized as macrophages infiltrating into the inflammatory sites of the fibrotic lesions. The time-course analysis of the lesions clearly revealed the close relationship between the infiltration of galectin-3-positive macrophages and fibrotic lesions induced by myocarditis. Increased serum level of galectin-3 correlated with the number of cardiac galectin-3 positive cells. These findings indicate galectin-3 expression might be a useful indicator of cardiac fibrotic degeneration in acute myocarditis following viral infection. Further studies are warranted to determine whether the serum galectin-3 might be useful and early diagnostic method for cardiac degeneration of myocarditis in humans.

## Supporting information

S1 Fig The evaluation of inflammatory response was graded as 0 = noe, 1 = mil, 2 = moderate, and 3 = severe as shown in the picture. Scale bar = 50 μm.
(PDF)

S1 Checklist Arrive guideline checklist. (PDF)
