# Supplementary material for: Time-course analysis of cardiac and serum galectin-3 in viral myocarditis after an encephalomyocarditis virus inoculation
Source: PLoS One. 2019 Jan 23;14(1):e0210971. doi: 10.1371/journal.pone.0210971 (PMC6343901; doi:10.1371/journal.pone.0210971)
Supplement: S1 Fig — Scale bar = 50 μm. (PDF) [file pone.0210971.s001.pdf]

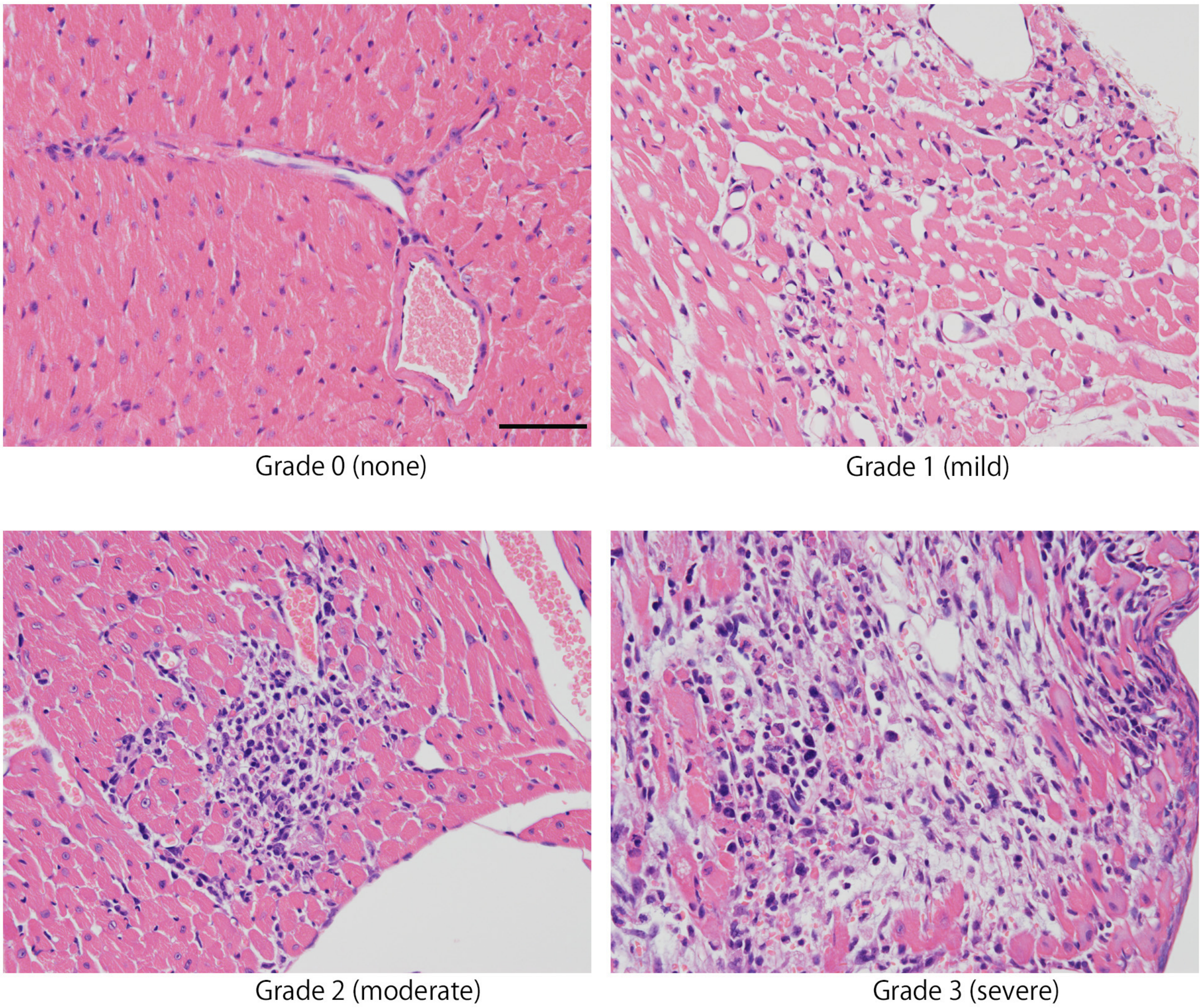

Figure S1. The evaluation of inflammatory response was graded as 0= none, 1= mild, 2= moderate, and 3= severe as shown in the picture. Scale bar = 50 µm.
